# Impact of Different Analytic Approaches on the Analysis of the Breast Fibroglandular Tissue Using Diffusion Weighted Imaging

**DOI:** 10.1155/2017/1094354

**Published:** 2017-03-02

**Authors:** Yoon Jung Choi, Jeon-Hor Chen, Hon J. Yu, Yifan Li, Min-Ying Su

**Affiliations:** ^1^Department of Radiology, Kangbuk Samsung Hospital, Sungkyunkwan University School of Medicine, Seoul, Republic of Korea; ^2^Center for Functional Onco-Imaging, University of California, Irvine, CA, USA; ^3^Department of Radiology, E-Da Hospital and I-Shou University, Kaohsiung, Taiwan

## Abstract

*Purpose*. This study investigated the impact of the different region of interest (ROI) approaches on measurement of apparent diffusion coefficient (ADC) values in the breast firbroglandular tissue (FT).* Methods*. Breast MR images of 38 women diagnosed with unilateral breast cancer were studied. Percent density (PD) and ADC were measured from the contralateral normal breast. Four different ROIs were used for ADC measurement. The measured PD and ADC were correlated.* Results*. Among the four ROIs, the manually placed small ROI on FT gave the highest mean ADC (ADC = 1839 ± 343 [×10^−6^ mm^2^/s]), while measurement from the whole breast gave the lowest mean ADC (ADC = 933 ± 383 [×10^−6^ mm^2^/s]). The ADC measured from the whole breast was highly correlated with PD with *r* = 0.95. In slice-to-slice comparison, the central slices with more FT had higher ADC values than the peripheral slices did, presumably due to less partial volume effect from fat.* Conclusions*. Our results indicated that the measured ADC heavily depends on the composition of breast tissue contained in the ROI used for the ADC measurements. Women with low breast density showing lower ADC values were most likely due to the partial volume effect of fatty tissues.

## 1. Introduction

Mammographically dense areas of the breast differ histologically from nondense areas: they have greater proportions of stroma and/or epithelium and smaller proportions of fat [1, 2]. The breast stromal microenvironment is composed of extracellular matrix (ECM) and different cell types, including endothelial and immune cells, fibroblasts, myoepithelial cells, and adipocytes, which are all capable of modulating epithelial function [3] and mammary duct morphogenesis [4]. It is noted that epithelial-stromal interactions play a crucial role in cancer initiation, progression, and invasion [5, 6]. Mammographically dense tissues are also associated with increased collagen-1 deposition in the stromal tissue [7]. Higher collagen levels in the mammary gland increase tumor formation and invasive behavior [8]. Mammographic density is a moderate risk factor for breast cancer, and studies of both epithelial and stromal components are important in understanding the association with breast cancer risk. New imaging methods are used to investigate the histological and functional aspects of the dense tissue.

DWI has gained popularity and become a complementary diagnostic tool for evaluating suspicious breast lesions in clinical breast MRI examination. DWI provides the opportunity to evaluate the microstructural information of the breast tissue, which may play a role in the pathogenesis of breast cancer. Additionally, DWI has also been used for the assessment of normal breast tissue [9–15]. Currently, the mainstream imaging modality for assessing breast density is 2D mammography, which has several fundamental limitations such as tissue overlap, the inconsistent positioning of the woman, and the inconsistent degree of compression. Magnetic resonance imaging (MRI) [16–18], on the other hand, is a great imaging modality for the volumetric measurement of breast density. Although traditional MRI methods cannot provide functional information of dense tissues, diffusion weighted imaging (DWI) can provide additional biologic information of different breast tissue components by depicting differences in the microscopic mobility of water molecules in normal or diseased tissues [19]. Unlike Brownian motion in a fluid, the freedom of water diffusion in tissues depends heavily on its interactions with the adjacent components, such as tissue type/arrangement, macromolecules, collagen fibers, cellular density, and the integrity of the cellular membranes. The movement of water molecules is known to be more restricted in tissues with high cellular density and less restricted in tissues with low cellular density [20]. Applying to the breast, DWI has the potential to elucidate the biophysical properties of the dense tissue. However, since the breast is composed of fatty tissue and fibroglandular tissue that have different diffusion coefficients, the measured ADC may vary with the fat content. This is a problem that needs to be resolved before the ADC can be used to investigate other cell types.

This study aimed to investigate the association of MR-based percent breast density with the apparent diffusion coefficient (ADC) acquired from DWI and to assess the impact of fatty tissue on measured ADC values. The breast consists of two major tissue components: fibroglandular tissue and fatty tissue. Lipid molecules are much larger than water molecules, and fatty tissues have a lower ADC value than fibroglandular tissues do [21]. In this study, we compared the ADC measured from the whole breast as well as from segmented fibroglandular tissues using different analysis approaches. These approaches allowed us to examine how the contamination of fatty tissue would affect the ADC values. We also investigated the associations between the measured ADC with the percent density analyzed from the whole breast.

## 2. Materials and Methods

### 2.1. Patients

Breast MR studies from 45 women (mean age 49 years, range 32–79 years) diagnosed with unilateral breast cancer who had T1-weighted imaging and DWI were retrospectively reviewed. These patients were from the diagnostic setting of our clinical patients. The study was approved by our institution's Institutional Review Board and the requirement of informed consent was waived. Seven women were excluded due to different breast coverage between ADC and T1 weighted images (*N* = 3), poor ADC imaging quality (*N* = 2), and the T1 weighted series not covering the entire breast (*N* = 2). The remaining 38 patients were analyzed. Out of these 38 patients, 30 had invasive ductal cancer, 5 had ductal carcinoma in situ, 2 had infiltrating lobular cancer, and one had invasive papillary cancer. Thirty-three patients received operations and had pathology-measured tumor size information (mean size 19.9 mm, range 1–60 mm). All 38 women had normal contralateral breasts without any symptoms or suspicious imaging findings, and only the normal breasts were used in this study. This study was approved by the Institutional Review Board (IRB) of our institution.

### 2.2. MR Imaging Acquisition

Breast MR imaging was acquired with a dedicated 7-channel breast coil in a 3.0T MR scanner (Achieva; Philips Medical System, Bothell WA, USA). Non-fat-suppressed, non-contrast-enhanced T1-weighted imaging (T1WI) in axial section was acquired first covering the entire breast bilaterally, followed by the dynamic contrast-enhanced (DCE) MR imaging. Diffusion weighted imaging was acquired after DCE-MRI. The imaging sequence and imaging parameters for the nonenhanced T1WI were spin echo, axial section, TR/TE 620/10 msec, matrix 332 × 332, field of view 200 × 340 mm, slice thickness 3 mm, and gap 1 mm. The images acquired from the nonenhanced T1WI were used for the segmentation of the whole breast and fibroglandular tissue. DWI was acquired in axial sections, utilizing a short tau inversion-recovery imaging (STIR) and a single-shot, spin echo, and echo planar imaging (SE-EPI) sequence with parameters: repetition time/echo time 3265/54 msec, flip angle 90 degrees, real *k*-space matrix 108 × 98, reconstruction matrix 288 × 288, field of view 350 × 350, in-plan spatial resolution 3.2 × 3.5 mm, slice thickness 4 mm, gap 1 mm, number of averages 2, and two b-factors 0, and 1000 s/mm^2^. STIR was used to achieve fat suppression effect. In total, 35 slices were acquired for DWI. The total scanning time of breast MRI, including positioning, was about 20 minutes.

The T1WI and the generated ADC images by the MR console were transferred to a personal computer offline in DICOM format for further analyses.

### 2.3. Imaging Processing and Analysis

#### 2.3.1. Breast Segmentation and Measurement of Percent Breast Density

The MR density measurement was done by using a well-established, template-based automatic segmentation method [22]. This automatic method has been shown to be very accurate compared to the radiologist's manual segmentation, revealing a percent difference ranging from 0.02% to 2.52% with a mean of 1.03%  ±  1.03% for the fibroglandular tissue segmentation [22]. With this method, the chest body region on a middle slice was used as the template. Within the chest template, an initial V-shape cut using three body landmarks (thoracic spine and bilateral boundary of the pectoralis muscle) was performed to determine the posterior lateral boundary of the breast. The chest template was mapped to each subject's image space to obtain a subject-specific chest model for exclusion. The chest and muscle boundaries determined on the middle slice were used as a reference for the segmentation of adjacent slices, and the process continued until all slices with breast tissue were segmented. The combined nonparametric nonuniformity normalization (N3) algorithm and fuzzy-*C*-means (FCM-) based algorithm were used to correct the signal intensity inhomogeneity [23], and *k*-means clustering (*k* = 6) was used to separate the fibroglandular tissue (the lower three intensity clusters) and the fatty tissue (the higher three intensity clusters). Percent density (PD) was calculated as the ratio of the fibroglandular tissue volume over the breast volume × 100%.

#### 2.3.2. ADC Pixel Distribution Analysis

**Figure 1:**
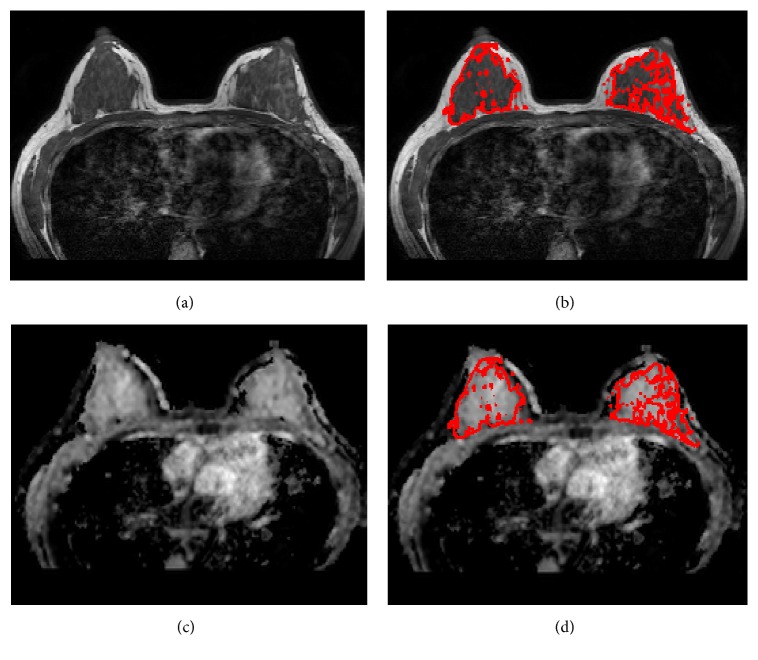
A 34-year-old woman with left breast cancer. (a) Non-fat-sat T1 weighted image from a central slice. (b) T1WI with segmented fibroglandular tissue mask. (c) The coregistered ADC map matching the T1WI. (d) ADC map with segmented fibroglandular tissue mask.

Using SPM (Statistical Parameter Mapping) package (Wellcome Department of Imaging Neuroscience; London, UK) in Matlab (Mathworks, Natick, MA, USA), all ADC maps were coregistered to the respective T1W images by affine transformation and normalized mutual information to have the same image-matrix size as that of T1W. The fibroglandular tissue masks from breast segmentation were then transferred onto the ADC maps and utilized as the region of interest (ROI) for ADC analysis. The pixels with unreasonable ADC values, defined as either below 100 × 10^−6^ mm^2^/s (considered as noise) or above 3200 × 10^−6^ mm^2^/s (approaching the self-diffusion value of free-water at body temperature), were excluded from the analysis.  Figure 1 illustrates a case that shows the segmentation of the fibroglandular tissue and the coregistration with the ADC map. The ADC histogram for each subject was generated using 32 equal-sized bins centered from 100 to 3200 [×10^−6^ mm^2^/s].

#### 2.3.3. ADC Values Acquired Using Different Methods

**Figure 2:**
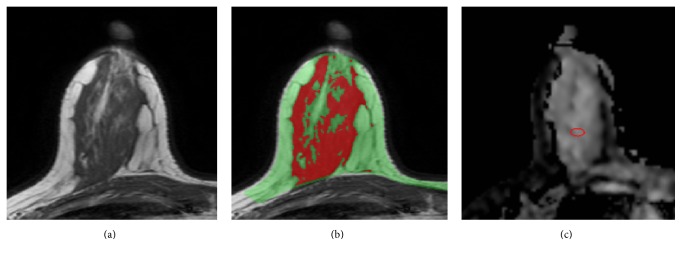
A 37-year-old woman with left breast cancer. (a) T1WI of the right breast from a central slice. (b) The segmented breast (green) and the fibroglandular tissue (red). (c) A small ROI is manually placed in the ADC map within the homogeneous fibroglandular tissue area. The percent density measured in the right breast is 24.8%. The mean ADC measured using 4 different methods are ADC_WB_ = 874, ADC_WF_ = 1250, ADC_SF_ = 1373, and ADC_SR_ = 1682 (×10^−6^ mm^2^/sec).

To investigate how fatty tissue would impact the measurement of ADC of the fibroglandular tissue, the ADC was measured using four different approaches: (1) averaged from the whole segmented breast (ADC_WB_); (2) from the segmented fibroglandular tissue of all slices in the whole breast (ADC_WF_); (3) from the fibroglandular tissue on a single slice from the central region of the breast (ADC_SF_); and (4) from a manually placed small ROI on the homogeneous fibroglandular tissue of the central slice (ADC_SR_). The central slice was selected by an experienced radiologist, who viewed all the axial section T1W images from each subject and chose a central slice that contained the most abundant fibroglandular tissue. The radiologist then manually placed an ROI within the homogeneous fibroglandular tissue, with a mean area of 28.1 ± 13.0 mm^2^ (range 10.7–66.1 mm^2^). An example of the manual ROI placement is shown in Figure 2. The results of ADC measured from these four methods were correlated with PD.

### 2.4. Statistical Analysis

Mean ± standard deviation (STD) of PD and ADC of the whole cohort were calculated. Pearson's correlation was used to correlate PD and ADC measured with different methods. The intraclass correlation was used to correlate the ADC measurements with different ROI methods. Student's *t*-test was used to compare ADC values acquired from different methods.

## 3. Results

### 3.1. Distribution of PD and ADC

The mean ± STD of PD for the 38 women was 14.8 ± 14.4% (range 2.2%–51.6%). Twelve of the women had very fatty breasts, with PD < 5%. Five women had dense breasts, with PD >30%. The mean ± STD of ADC values measured using the four methods is shown in Table 1. As noted, the ROI-measured mean ADC on a single central slice (ADC_SR_) had the least amount of fat contained in the ROI and the highest mean ADC (1839 ± 343 [×10^−6^ mm^2^/s]). Since ADC measured from the whole breast tissue (ADC_WB_) had the highest amount of fat, the mean ADC value was the lowest (933 ± 383 [×10^−6^ mm^2^/s]). The ADC measured from the FT of the whole breast and the single central slice were ADC_WF_ = 1243 ± 295 and ADC_SF_ = 1385 ± 304 [×10^−6^ mm^2^/s].

### 3.2. Correlation of PD with ADC

**Figure 3:**
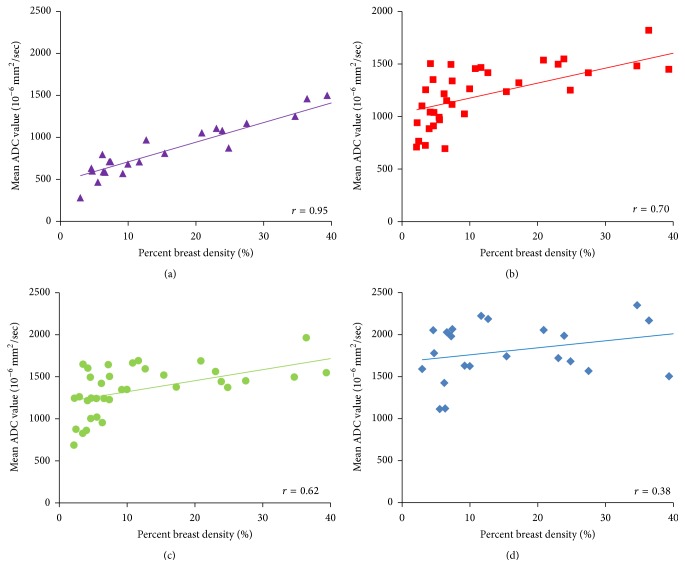
Correlation of the percent density (PD) with ADC measured using four different methods. (a) ADC measured from the whole breast (ADC_WB_) shows a high correlation with PD, with *r* = 0.95. (b) ADC measured from the segmented fibroglandular tissue in the whole breast (ADC_WF_) shows a moderate correlation with PD, with *r* = 0.70. (c) ADC measured from the segmented fibroglandular tissue in a single central slice (ADC_SF_) shows a moderate correlation with PD, with *r* = 0.62. (d) ADC measured from a small manually placed ROI (ADC_SR_) shows a weak correlation with PD, with *r* = 0.38. From (a) to (d), as the fat content decreases, the ADC increases and shows worse and worse correlation with the whole breast PD.

**Figure 4:**
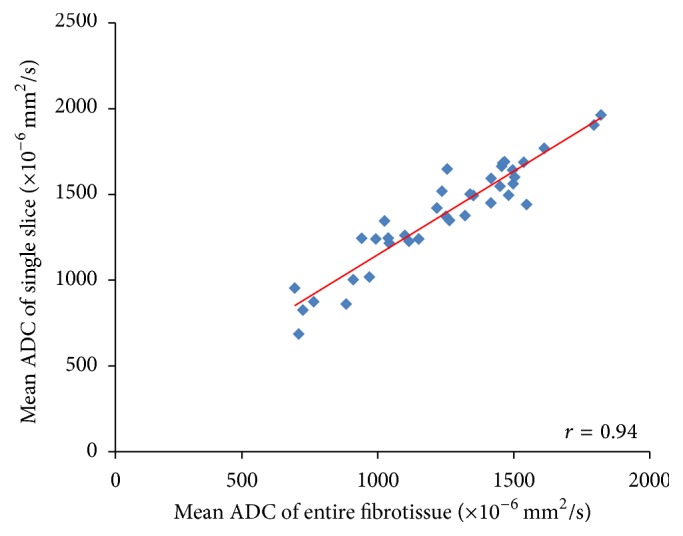
The correlation of ADC measured from the fibroglandular tissue in a single central slice (ADC_SF_) with the fibroglandular tissue in all slices in the whole breast (ADC_WF_).

**Figure 5:**
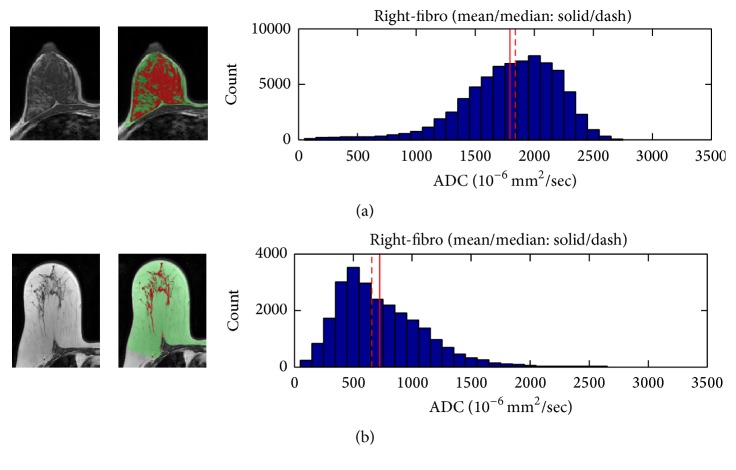
The T1WI, segmented fibroglandular tissue, and histogram of ADC values measured from the fibroglandular tissue of the whole breast. (a) A 45-year-old woman with PD = 47.6% and ADC_WF_ = 1795 × 10^−6^ mm^2^/sec. (b) A 58-year-old woman with PD = 3.5% and ADC_WF_ = 724 × 10^−6^ mm^2^/sec.

**Figure 6:**
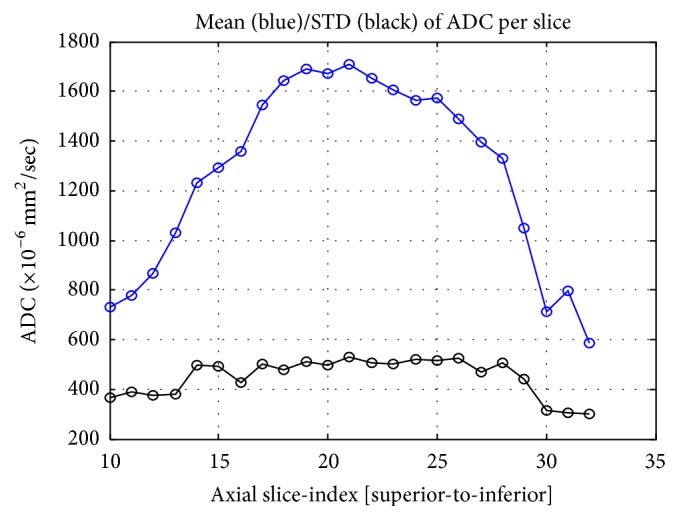
The slice-to-slice profile of the mean ADC value measured in the fibroglandular tissue of different imaging slices in a craniocaudal order. The number of fibroglandular tissue pixels increases from the superior slices to the central slices and then gradually decreases towards the inferior slices. The fat partial volume effect is higher in the peripheral than the central slices, which leads to the variation in the ADC profile.

The correlation of PD with ADC measured from different methods is shown in Figure 3. Of the 38 subjects, the radiologist could only place ROI within the fibroglandular tissue area in 25 women and measured the ADC values. The remaining 13 subjects could not be measured using this method due to having fatty breasts or mixed breast patterns. The ADC measured from the whole breast (ADC_WB_) was strongly correlated with PD, with *r* = 0.95 (Figure 3(a)). In contrast, the ADC measured from the manual ROI (ADC_SR_) was not strongly correlated with PD, with *r* = 0.38 (Figure 3(d)). The correlation coefficients using ADC values measured from the whole fibroglandular tissue volume (ADC_WF_) and from the fibroglandular tissue in a single slice (ADC_SF_) showed moderate relationships (*r* = 0.70 and *r* = 0.62, resp.) (Figures 3(b) and 3(c)). Although ADC_WF_ and ADC_SF_ were measured from the coregistered fibroglandular tissue from the segmented FT mask, there was inevitably partial volume effect with fat, especially in the boundary of the FT mask adjacent to the fatty tissue. Overall, ADC_SF_ and ADC_WF_ measured from a single slice and from the multislices in the whole breast had a strong correlation using Pearson correlation and intraclass correlation (both *r* = 0.94) (Figure 4). However, as noted, the ADC values from the single slice measurement were consistently higher compared to the ADC measured from the whole fibroglandular tissue volume (*p* = 0.04). The *p* values for the comparison of ADC measured using four different methods are listed in Table 2 and show a statistically significant difference for each pair of comparison.  Figure 5 shows the histogram distribution of ADC values from a 45-year-old woman with a high breast density (PD = 47.6%) and high ADC of 1795 × 10^−6^ mm^2^/sec versus a 58-year-old woman with a low breast density (PD = 3.5%) and low ADC of 724 × 10^−6^ mm^2^/sec.  Figure 6 shows a case demonstrating the slice-to-slice variability of the measured mean ADC values in a craniocaudal order. It was obvious that the central slices containing more fibroglandular tissue had higher ADC values, presumably due to less partial volume effect from fatty tissue compared to peripheral slices. As the content of fatty tissues towards the peripheral slices increased, the measured ADC values gradually decreased.

### 3.3. ADC Values Stratified by Breast Density

**Figure 7:**
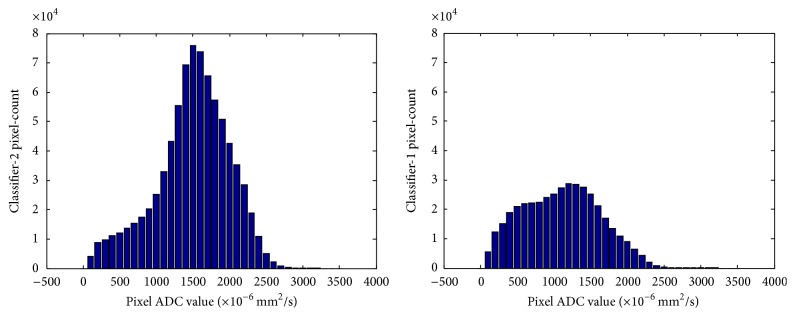
Comparison of the histogram distribution shape of the two density groups.

**Figure 8:**
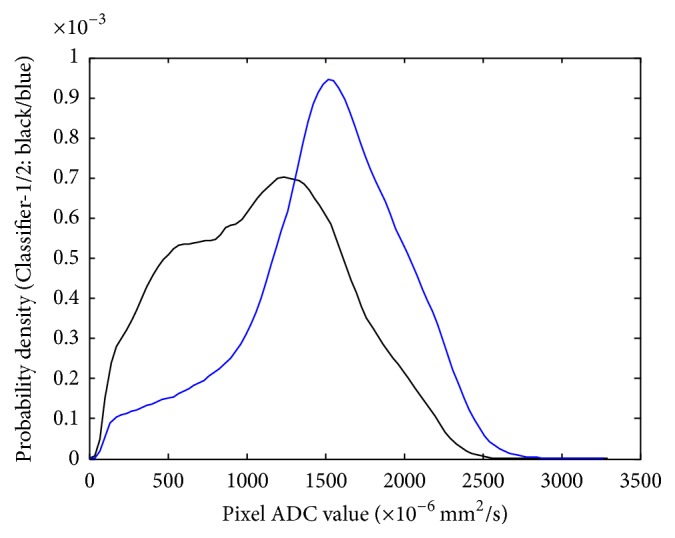
The probability density curves of the two density groups. Note that women with higher density (blue color) tended to have higher probability of high ADC values.

The mean and standard deviation of ADC of each patient based on the four different ROI methods between the 2 groups of patients based on the 10%-PD-cutoff were analyzed and showed statistically significant difference of all the comparisons in the two density groups (Table 3). The mean ADC of the whole fibroglandular tissue of women with higher PD (*N* = 16) was higher than that of women with lower PD (*N* = 22) (1498.7 ± 166.0 [×10^−6^ mm^2^/s] versus 1149.0 ± 220.2 [×10^−6^ mm^2^/s]). Overall, women with lower PD tended to have evenly distributed pixel-counts among different histogram bins, as opposed to a bell-shaped histogram observed in women with higher PD (Figure 7). The probability density curve (Figure 8) showed that women with higher PD tended to have higher probability of ADC higher than 1500 [×10^−6^ mm^2^/s] than women with lower PD. There was no significant correlation between any ADC summary statistics and age.

## 4. Discussions

In this study, we used a well-developed, template-based autosegmentation algorithm [22] to segment the fibroglandular tissue. We then mapped the segmented fibroglandular tissue onto the ADC images through coregistration to measure ADC values using different ROI methods. Since fatty tissues are large molecules and have low ADC, women with higher breast density have less fat and thus have higher ADC_WB_ values measured from the whole breast. Our results were consistent with those of other studies, which also showed that the ADC value of normal breast tissue was lower in predominantly fatty breasts than in dense breasts [10, 12], and increased breast density was strongly associated with increased ADC (*p* ≤ 0.0001) [15]. Another study, however, noted no differences in ADC measures among the different breast density categories [24]. We also showed that the ADC measured from the fibroglandular tissue could vary substantially depending on how the ROI was chosen. We believed that both technical/methodological issues and histological characteristics of different breast tissues might account for the differences in the reported ADC values [9, 12, 14, 15].

The ADC value was lower in fatty breasts than in dense breasts, which was expected due to the different amount of fat content [12, 15]. The results in this study showed that the ADC measured from the manually placed fibroglandular tissue ROI was the highest, while the ADC measured from the whole breast was the lowest; in addition, the ADC values measured from the single slice containing the largest amount of fibroglandular tissue were significantly higher than the ADC values measured from the whole fibroglandular tissue volume. By using the four different analysis methods to measure ADC, it was clear that the correlation of ADC with PD became increasingly weaker when the measurement of ADC went from the whole breast to a well-defined ROI in the fibroglandular tissue area. The correlation of PD with ADC measured from a small homogeneous fibroglandular ROI was weak (*r* = 0.38), but the other methods showed stronger correlation, suggesting that the “pseudocorrelation” most likely came from the inclusion or contamination of fatty tissue which would lower ADC values in women with low PD. Another evidence of the contribution of the fatty tissue to the measured ADC was the slice-to-slice profile shown in Figure 6, which demonstrated decreasing ADC, especially when close to the edge slices. Our findings concurred with a literature report that concluded that ADC was generally higher in the central breast region, which may be partially due to larger areas of fibroglandular tissue in this region, resulting in less fat partial volume effect [11].

Thus, optimal fat suppression technique is important for accurate measurement of ADC for evaluation of the fibroglandular tissue or suspicious lesions [12, 24]. When comparing fat-suppressed versus nonfat-suppressed DWI, systematic underestimations of ADC for the normal breast tissue on nonfat-suppressed DWI were noted, which is likely due to the intravoxel contribution of the fat signal [24]. We adopted a STIR method for fat suppression in DWI. STIR-based methods are more resistant to the main magnetic field inhomogeneity and thus hold more promise than other techniques, such as CHEmical-Shift Selective (CHESS), in the evaluation of the breast using DWI [25]. However, unless an adiabatic radiofrequency (RF) pulse is used, STIR is sensitive to the spatial nonuniformity of the applied RF pulse. If the strength of the RF pulse varies, the tip angle of the inversion pulse and the quality of the fat suppression will differ across the images. In general, fat suppression quality is highly dependent on factors such as magnetic field homogeneity, coil sensitivity, and air-tissue susceptibility differences and can provide variable results for breast imaging [24]. Nevertheless, even with high-quality conventional fat suppression techniques used in DWI, triglyceride signal, including glycerol backbone (4.07 ppm and 4.23 ppm) and olefinic acid (5.3 ppm), which appear near the water peak, cannot be suppressed [26].

Intravoxel fatty component is also an issue. A study conducting histological examination of biopsied specimens from the dense area of 59 healthy women showed that the average fat component in the dense tissue is 29.7% [1]. The significant fatty component in the fibroglandular tissue, when not suppressed completely, might contribute to the reduction of ADC. Even in our study, which used a carefully defined ROI method, two women with low breast density showed mean ADC values lower than 1200 [×10^−6^ mm^2^/s]. It is unknown whether this low ADC resulted from real histological contribution or simply from fatty tissue contamination. In clinical practice, this may cause diagnostic confusion because the low measured ADC values in the normal fibroglandular tissue due to fat contamination may look similar to low ADC values in a malignant lesion due to a high cellular density. This phenomenon may be even more severe in women with very fatty or heterogeneous breast pattern, as in case of the 13 subjects who could not be confidently measured in this study. It is expected that the correlation between the percent density and the measured ADC decreases as the examined ROI becomes smaller.

Since DWI is currently a popular diagnostic tool for different body parts and fatty tissue is one of the major tissue components in the body, careful definition of ROI is important to having an accurate and reliable ADC measurement. However, in real clinical practice, it is sometimes not possible due to the poor spatial resolution of the acquired images. For example, DWI is not reliable for differentiating between benign and malignant lymph nodes in patients diagnosed with rectal cancer due to the potential contamination of the adjacent mesorectal fat [27]. A recent study using magnetic resonance imaging (MRI) which showed invasive breast cancer preferably and predominantly occurs adjacent to breast adipose tissue. Of 294 patients, 291 had DCE-MRI discernible invasive breast tumors located at the interface between fibroglandular and adipose tissues, regardless of the tumor size, type, receptor status, or breast composition [28]. Similarly, our unpublished results from a separate dataset (JH Chen, UC Irvine) also confirmed their results. In our study of 122 Asian women diagnosed with unilateral breast cancer, subjective assessment based on an experienced breast radiologist's interpretation showed that 85 patients (85/122 = 69.7%) had more than 50% of the tumor margin contacting with the fatty tissue. Twenty-four women (24/122 = 19.6%) had less than 50% of the tumor margin contacting with the fatty tissue. Only thirteen patients (13/122 = 10.7%) had tumor totally included in the fibroglandular tissue. With objective quantitative measurement, of the 122 patients, only 2 women had tumor totally included in the fibroglandular tissue. The remaining 120 patients (120/122 = 98.4%) had breast lesion contacting with the adjacent fatty tissue. As the ADC results in Figure 6 show for fibroglandular tissue in different slices, it is reasonable to postulate that the fat partial volume effect may be higher for small tumors than for large tumors. A robust objective ROI method to define or segment the tumor area is thus very important.

In addition to the fat partial volume effect, histological characteristics of breast tissues may lead to intrinsic differences in the measured ADC. It was noted that normal breast tissue does not have a uniform (homogeneous) ADC, which can be partially attributed to the anisotropy of water diffusion in normal breast tissue [11]. Histologically, the branching mammary ducts and associated periductal fibrous stroma converge from the peripheral breast tissue towards the nipple; thus the water molecules may follow a less restricted path and diffuse preferentially along or parallel to the ducts [11]. A study [10] comparing the ADC values in different breast areas noted that the retroareolar area has a higher ADC value compared to that of the upper outer quadrant area due to having more fibroglandular tissue and higher concentration of mammary ducts. Since women with higher PD normally have more retroareolar fibroglandular tissue compared to women with lower PD, the retroareolar area may contribute more pixels with high ADC, leading to higher mean ADC values.

Histologically, a higher percent mammographic density (MD) is associated with a significantly greater nuclear area of both epithelial and nonepithelial (stromal) cells, a greater proportion of collagen, and a greater area of glandular structures [7]. The relative concentration of epithelial cells in high density areas, compared with low density areas, was found to be 12.3 in terminal duct lobular units and 34.1 in ducts [29]. Histological evidence from mastectomy specimens also showed that areas of higher mammographic density have higher glandular counts and greater proportion of smaller and less complex glands [2]. Although the stroma is the main increased histological component in women with high MD, the packing of the collagen-dense stroma is not increased [2]. Although increased amounts of different tissue components in the dense tissue may impact the measurement of ADC in different ways, it is generally believed that increased ADC in women with high breast density is possibly due to increased water content from the increased cellularity and secretion activities in the fibroglandular tissue [9, 14].

Limitations of our study include the small number of subjects and the consequent need to validate results using more subjects with different breast patterns. In addition, our subjects have an increased risk of developing breast cancer in the contralateral breast, which may confound the properties of the fibroglandular tissues being examined. Furthermore, a small gap of 1 mm existed between adjacent slices for both T1 and DWI. Although the coregistration processes used interpolation to fill in the gap, this may suffer from a small interpolation error. A true volumetric acquisition for both T1WI and DWI will be optimal for the coregistration process used to transfer segmented tissue masks to the ADC maps.

## 5. Conclusions

In conclusion, we performed a systematic analysis to investigate the ADC values measured in the normal breast by coregistering ADC maps with segmented breast and fibroglandular tissues. The results showed that the accuracy and reliability of the measurement of ADC are heavily dependent on composition of tissues included in the ROI analysis. While the ADC measured from the whole breast showed a high correlation with the overall percent density, the correlation became weaker with more carefully selected homogeneous fibroglandular tissue ROI's. Even in the carefully segmented fibroglandular tissues, there is inevitable fat contamination due to partial volume effects and moderate correlations with the percent density. We used the fat-suppressed STIR DWI sequence in this study; when a nonfat-suppressed DWI is used, the effect is expected to be even more pronounced. The results from different approaches of analysis in this study suggested that lower ADC in women with low breast density is most likely due to the partial volume effect of fatty tissues. Although it will be interesting to use DWI to investigate different tissue components in the fibroglandular tissue that are associated with breast cancer, the fat contamination problem must be resolved first.

## Figures and Tables

**Table 1:** Mean ± STD and range of ADC values measured using four different methods.

	Mean ± STD ADC [×10^−6^ mm^2^/s]	Range of ADC [×10^−6^ mm^2^/s]
ADC_WB_	932.8 ± 382.6	282.1–1692.5
ADC_WF_	1243.0 ± 294.5	693.6–1820.8
ADC_SF_	1385.1 ± 303.5	685.7–1964.2
ADC_SR_	1839.3 ± 343.2	1114.9–2350.1

**Table 2:** *p* values for the comparison of ADC measured using four different methods.

	ADC_WB_ versus ADC_WF_	ADC_WB_ versus ADC_SF_	ADC_WB_ versus ADC_SR_	ADC_WF_ versus ADC_SF_	ADC_WF_ versus ADC_SR_	ADC_SF_ versus ADC_SR_
*p* value	0.00132953	1.10635*E* − 05	1.31294*E* − 11	0.041858	5.86685*E* − 09	2.32358*E* − 06

**Table 3:** Comparison of ADC histogram distribution in the low and high breast density groups.

	Low density group		High density group		Corrected *p* values
	Mean	SD	Mean	SD	
ADC_WB_ (×10^−6^ mm^2^/sec)	603.0	138.3	1191.9	302.0	**0.0000032**
ADC_WF_ (×10^−6^ mm^2^/sec)	1149.0	220.2	1498.7	166.0	**0.00036**
ADC_SF_ (×10^−6^ mm^2^/sec)	1316.1	204.8	1620.4	173.5	**0.00083**
ADC_SR_ (×10^−6^ mm^2^/sec)	1674.0	349.1	1969.2	287.0	**0.035**
